# Innovating Novel Online Social Spaces with Diverse Middle School Girls: Ideation and Collaboration in a Synchronous Virtual Design Workshop

**DOI:** 10.1145/3491102.3517576

**Published:** 2022-04-29

**Authors:** Catherine Grevet Delcourt, Linda Charmaraman, Sidrah Durrani, Quan Gu, Le Fan Xiao

**Affiliations:** Wellesley College, Computer Science Department; Youth, Media & Wellbeing Research Lab, Wellesley Centers for Women; Teachers College, Columbia University, Department of Human Development; Wellesley College; Wellesley College

**Keywords:** Participatory design, social media, synchronous communication, adolescent girls, confidence, collaboration, small-group facilitation

## Abstract

Leveraging social media as a domain of high relevance in the lives of most young adolescents, we led a synchronous virtual design workshop with 17 ethnically diverse, and geographically-dispersed middle school girls (aged 11–14) to co-create novel ICT experiences. Our participatory workshop centered on social media innovation, collaboration, and computational design. We present the culminating design ideas of novel online social spaces, focused on positive experiences for adolescent girls, produced in small-groups, and a thematic analysis of the idea generation and collaboration processes. We reflect on the strengths of utilizing social media as a domain for computing exploration with diverse adolescent girls, the role of facilitators in a synchronous virtual design workshop, and the technical infrastructure that can enable age-appropriate scaffolding for active participation and use of participatory design principles embedded within educational workshops with this population.

## 1 INTRODUCTION

Middle school (10–14 years old) marks a key transitional period in early adolescent identity and social development. Building a strong sense of belonging, learning empathy and their role as part of a larger community are key components of this developmental stage [38, 44]. This evolving social environment often marks early initiation in the usage of communication technologies as core aspects of our connected world today [29]. Recently, during the COVID-19 pandemic and era of physical distancing, Information Communication Technologies (ICT) have become even more essential parts of adolescents’ daily lives [10]. This period of early adolescence is also a turning point for girls in their self-concept as future creators and innovators. Girls’ confidence in their science and engineering abilities often wanes during adolescence [2, 3]. Fostering positive activities and experiences that promote creativity, confidence, and a sense of belonging can empower them in these disciplines [20, 27]. In HCI, *participatory design* (PD) practices with children aim to promote these competences through hands-on approaches to digital innovation [19] and an emphasis on the social and *intergenerational* contexts of these projects [50]. However, the particular needs, social, emotional and cognitive, of diverse middle school girls have not been directly tackled in these contexts, particularly in remote approaches where challenges and opportunities may be different than those of in-person experiences.

Leveraging social media, as a topic of high-relevance in the lives of middle school girls, we created a virtual synchronous participatory design workshop to embed our infrastructure and our discussion topics in ICTs. We led a 4-day remote summer workshop in 2021 with 17 diverse, geographically-dispersed U.S. based middle school students. Our informal curriculum structure addressed topics relating to social wellbeing, identities in science and engineering, and computational concepts related to social media. Through this workshop, we engaged students in online design activities, adapted from prior work during in-person design sessions with youth. In small groups led by facilitators, students envisioned and innovated novel online social application ideas to support positive interactions amongst adolescents. Our work extends best practices and understandings of virtual synchronous design workshops with youth by focusing on the socio-technical needs of underrepresented middle school girls.

Our contributions are:

1. A description of novel ideas for online social spaces generated by participants in the workshop.
2. An account of collaboration strategies for virtual synchronous design workshops with middle school girls.

## 2 RELATED WORKS

Our work is situated at the intersection of participatory design with youth, learning sciences, and social computing. We describe the prior work that has framed our workshop curriculum and our research goals, and explain the domain of social media as our highly relevant topic of discussion and design for our population.

### 2.1 Developing confidence for adolescent girls as digital creators

Until 10 years old, children tend to remain positive and confident about science learning [2, 3]. Vicarious learning experiences by observing others, particularly with role models one identifies with, have been shown to be especially effective for women compared to men [51]. Social and cultural feedback and implicit persuasion from one’s contexts can encourage or discourage girls from building efficacy in areas outside traditionally acceptable roles and career goals [25]. Particularly for girls of color, decisions about exploring a career in Computer Science are influenced by prior experience, interest, and sense of fit with their communities [30, 35]. Gender and racial stereotypes within STEM learning settings make it difficult for girls to develop a sense of belonging [33]. Thus, they are missing in representation as creators of digital information communication systems. It’s a perpetual cycle: self-efficacy often predicts women’s career choices, whereas self-efficacy is influenced by gendered career perceptions [6]. Self-efficacy plays a crucial role as a dynamic set of beliefs that an individual holds about her ability to perform a domain-specific task [5]. For instance, research has found a gender divide in defining the value-added domain of communication skills. Girls tend to view good communication skills as being adept at talking and listening, whereas boys are more likely to rate this as being adept at math and ICTs [16]. Additionally, boys have been shown to be more confident in their ability to speak to their classmates, explaining their points of view, and asking when they don’t understand something with known and unknown adults, compared to their female classmates [16]. Prior educational interventions have demonstrated that it is possible to shift this perception by increasing confidence and connecting their learning to present and future utility and relevance [20, 27].

### 2.2 Participatory design with middle school students

The philosophy of *participatory design* (PD), or communal participation for matters of collective importance, originates in the very basis of democratic politics [41]. Humanist approaches to cooperatively designing socio-technical design emerged in Europe and Scandinavia in the 1970s and, later in the 1980s in the US, influenced the early development of personal computing through engaging end-users as designers [19, 41]. The intent of PD, in the context of HCI, is to empower those most directly impacted by novel technologies to become creators of them. PD is particularly impactful for working with marginalized populations which has raised key issues around values, processes, and power dynamics [41]. With children, assistance by adults is often necessary, for example to aid with technical skills for prototyping, and the resulting power dynamic is of particular interest. Druin coined *Cooperative Inquiry*, a PD approach in which children are equal design partners, bringing their own expertise to the table [19]. Following Druin’s influential call for addressing the role of children in research, many methods and activities for youth have been developed in HCI [15, 19, 24, 41].

#### 2.2.1 Social context of design.

In PD with children, participation takes place in *intergenerational teams* between youth participants and adults overseeing the sessions [50]. An outcome of PD is often an impact on participants and researchers, through mutual learning [9]. A framework of intergenerational interactions developed by Yip et al. suggests that core collaboration strategies observed in these settings are: Facilitation “how much support and mediation takes place between the adults and children”, Elaboration “generating and mixing ideas together”, Design-by-Doing where “design activities take place” and Relationships which refers to rapport building [50]. This framework was developed for in-person design sessions and remains underexplored for intergenerational interactions in virtual contexts. Further, exploring age-appropriate PD methods for early adolescents, specifically the middle school girl population, where the shifting social context may impact the social dynamics of design sessions remains exploratory [21]. In fact, it is not well understood how the social context influences design and creativity tasks for early adolescents [22, 31].

#### 2.2.2 Activities in participatory design with youth.

Common activities with children in design partners and Cooperative Inquiry contexts are typically low-fidelity and low-tech, using familiar materials [48]. Some well-documented activities include: low-tech prototypes [17, 43]; post-it notes, hand sketching, comic boarding [36, 48]; or layered elaboration [46]. In addition to age-appropriate adaptations for conducting the design sessions themselves, research has also concerned itself with addressing the ethics of youth participation, such as ensuring that consent and transparency of research are understood by children, and with analyzing the outputs of design sessions [41]. Research has also concerned itself with the utilization and classification design outputs generated by children in PD contexts [15, 24]. The outcome of PD is not necessarily a final product. Many PD projects strive for a product, where youth inform the design process through active participation [26]. However, the outcome of PD can also be focused using design as a tool for discussing personal circumstances and giving participants a forum to express their concerns or desires.

#### 2.2.3 Distributed participatory design.

PD traditionally concerned itself with co-located contexts, such as amongst co-workers [45]. More recent work has explored how to enable co-design within distributed communities, or geographically-dispersed groups. Prior to the COVID-19 pandemic, these distributed co-design projects mainly focused on asynchronous interactions [45] and few explored the socio-technical needs of youth in distributed PD contexts. The Asynchronous Remote Communities (ARC) framework focused on older teens (e.g., ages 13–19 [7]) where technologies are available (Facebook, Slack, and Discord are not available to those younger than 13) and asynchronous communication may be suitable. Due to the recent context of physical distancing, emergent work has explored synchronous design sessions with younger children aged 7–11 [34]. In their framework, Lee et al. characterize the technical infrastructure for synchronous design workshops with youth according to four dimensions: Meeting, Sharing, Collaboration, and Design spaces [34]. They suggest utilizing familiar collaborative tools, such as PowerPoint and Zoom, as their primary mode of digital creation [34]. Another workshop with a population aged 12 to 14 years old conducted a distributed maker workshop and found that opportunities for distributed co-design were utilizing multi-channel communication for personalized support, adapting learning to topics of high-relevance, and allowing backchannel collaboration amongst facilitators [37]. Facilitation in distributed PD with youth may require more involvement than in-person [47]. Few projects have specifically explored the social needs of middle school girls in virtual synchronous participatory design contexts and the emergent practices of facilitators in distributed PD, particularly with children as design partners, remains an open area of research.

### 2.3 Social media as a topic of high-relevance for early adolescent girls of color

National studies conducted by Pew Research Center have found gender differences in social media use. For instance, girls are more likely to report being constantly online and to use Snapchat compared to boys [1]. Teen girls are more likely to post about a variety of topics, post selfies, and feel the need to disconnect or disengage from their social media than teen boys [1, 11]. Girls are also more likely to seek out online social spaces that are about such topics as health, wellness, and being a member of a subgroup, such as being a racial/ethnic minority [1]. To inform our local theory planning in our broader project on online wellbeing for early social media users, taking into account underrepresented middle school girl interests and needs regarding ICT, we analyzed survey data that is part of a larger NIH-funded study with 968 students in local middle schools [14]. The sample population was 45% boys, 53% girls, 2% non-binary and racially/ethnically diverse (52% White, 16% Latinx, 8% Black, 6% Asian, 4% Native American, 7% Biracial, 3% Middle Eastern, 4% Other). Twenty-five percent of students were in 6th grade, 21% in 7th grade, 23% in 8th grade, 16% in 9th grade and 15% in 10th grade. We conducted preliminary analyses to understand what social media-related topics were most relevant according to three demographic categories: gender, race, and grade level.

#### Race:

We found that non-White participants were most likely to have only talked to friends and never talked to a parent about their social media use compared to White participants, particularly regarding being hooked on devices and digital footprint. Non-White participants were also more likely to give advice to their peers about posting something that would get a lot of views.

#### Grade Level:

We found that older middle school students (i.e., 8th graders) were significantly more likely to talk to their peers about the following topics: hate speech, managing online drama, and comparing yourself to others. When older students give advice about using social media, they often focus on setting up profiles and posting photos. Younger students (i.e., 6th graders) kept more profile information private on social media than older students. Within middle school, students’ developmental needs in different grades can vary widely, from the transition out of elementary school and getting one’s first phone to getting ready for high school.

#### Gender:

Girls were naturally drawn to talking about social media with their peers. We found that compared to boys, girls were significantly more likely to talk to their peers about the following topics: hate speech, managing online drama, comparing yourself to others, and considering how your posts might make others feel. When girls give advice to their peers about using social media, they often focus on setting up profiles, posting photos, and avoiding drama.

Since girls are impacted as consumers of social media content in many unique ways, it is important to hear their voices as producers of social technology. Previous work on participatory design principles, techniques for developing confidence in adolescent girls, and our preliminary findings on how adolescent girls have demonstrated strong interest in their online social spaces and offering advice to peers all provide a strong rationale that an ICT innovation workshop, centered around social media use, would be highly suitable to middle school girls who are already invested in these social technologies.

Research questions:

RQ1: What types of design ideas are conceptualized by early adolescent girls for positive, supportive online social spaces?

RQ2: What are effective methods and tools to support collaborative design processes in virtual synchronous environments with early adolescent girls (11–14)?

## 3 METHODS

Our workshop is part of a larger longitudinal study on online well-being for early social media users. Following a social media app design pilot workshop in summer 2019 [13] and a virtual workshop in summer 2020 [12] focusing on identity and social wellbeing, we ran a virtual summer workshop for the third consecutive year with middle school students. Our goals for this workshop were to 1) explore social media innovation, and 2) create a safe and collaborative space for young adolescent girls to explore design ideas. Our focus on a middle school girl population expands on prior work by exploring age-appropriate and gender-specific collaborative tools, activities, and processes for communities of color.

### 3.1 Facilitator and team composition

Our team consisted of two PIs from interdisciplinary backgrounds, one from Computer Science and the other from Psychology and Positive Youth Development who facilitated the whole group activities and discussions. They had a project coordinator who handled recruitment, consent forms, and logistics. Together, they trained four small-group facilitators—a Masters student in Developmental Psychology, two undergraduate students, and a rising high school senior. We define facilitators following the established definition of “*a person responsible for planning and leading PD activities and for reporting the results to the rest of the design team or others*” [18]. Small groups were composed of 3 to 4 students and one facilitator. These sizes were determined by practical reasons that we were limited by the size of our organizational team (6 facilitators) and the number of students participating in the workshop. This also corresponds to recommendations for the group size of design sessions with youth [8]. Within groups, we prioritized homogenous of ages and grade levels. This was driven by our goal to foster a comfortable environment since our school-wide survey indicated that students in specific grades had significantly different experiences with cellphone usage and social media [14]. We will use the term *facilitator* for the 6 members of our organizational team who were trained and led the design sessions, *participants* for the youth who were present during the design sessions, and, following the terminology of PD, we will use the term *design partners* when referring to a group of individuals (facilitator and participants) involved in one design session [19, 50].

### 3.2 Participant recruitment, IRB, and ethics

Our recruitment included announcements by principals, institutional faculty/staff mailing lists, social media posts, and by word of mouth. Additionally, we reached out to over 32 organizations and educational institutions such as Black Girls Do STEM. We obtained 29 applicants from 5 states and used the following selection criteria for final invitations: a) applicants from a prior workshop waiting list, b) students from our partnering school, c) girls from underrepresented backgrounds (e.g., racial/ethnic minorities). Parents of the minors signed consent forms and media releases, and students provided assent to participate. Media releases included permission to use participant images, artwork, and de-identified audio and Zoom video recordings for future reports.

Due to the sensitive nature of our workshop topics, we emphasized inclusion and respect for others through a code of conduct established at the beginning of the workshop. The code of conduct outlined respectful behavior such as being mindful of the diversity and experiences of other students, being open to feedback, and not bullying their peers or using any offensive language. Additionally, conduct for Zoom was outlined in which participants were requested to keep their cameras on, and mics of when not participating. Drawing from previous research on discussing sensitive topics with adolescents, facilitators used a mix of open-ended and closed-ended questions and prioritized on building trust and rapport with the students [4]. Students were also reassured that all information shared would be de-identified in future dissemination of the work.

### 3.3 Workshop structure

The goals of our workshop were to a) discuss and re-imagine positive social media experiences and technology-related careers for underrepresented adolescent girls; b) implement an informal virtual workshop that incorporated participatory design philosophy and methods as one of the key activities; and c) explore and evaluate ways to facilitate these methods for maximum engagement for participants of different ages/comfort levels and future implementation. We selected an overarching theme for our workshop on a topic of high-relevance for diverse middle school girls: exploring the design of novel positive social online spaces.

#### 3.3.1 Digital ecosystem.

To foster a comfortable and safe environment for middle school girls, we utilized the panoply of remote tools that became familiar and widespread during the remote educational setting of the COVID-19 pandemic, and adapted offline participatory techniques with youth to this remote context. The workshop was conducted synchronously through Zoom for video/audio conferencing. We also heavily leveraged Google Suite as a collaborative environment that was available and familiar to our population [42]. All students had access to a Google Site that contained the daily workshop schedules, Zoom links, and Google Slide links for the small group design and discussion sessions. Seesaw classroom was the site used for activities and assignments, as well as a digital journal for students to write down daily reflections. Seesaw activities provided private spaces for personal reflections, often utilized in design processes such as innovator’s notebooks [49]. Google Slide was used in the small group breakout room design sessions, and students were given the option to edit directly on the slides if they wished to and had the means to do so.

#### 3.3.2 Structure of the design sessions.

To contextualize the design sessions within our broader educational structure, we describe the activities from Day 1 that did not include a design session, and provide a detailed agenda in the Appendix. The workshop included discussions of the design innovation process, social media usage and habits particularly as it is experienced by young girls, female guest speakers in the tech industry, and design sessions facilitated in small age-based groups. Design sessions followed the early phases of the user-centered design process starting with empathizing, then brainstorming and ideation which culminated in a final project presentation. Our workshop structure and co-design activities were inspired by best-practices for intergenerational participatory design techniques [48]. Our goal was not to obtain a functional prototype by the end of the workshop, but rather create a mutual learning environment in which participants felt supported and engaged. Design session were structured in a way to be conducted remotely with minimal technical resources and assistance. To cater to the age of participants, design sessions were less than an hour long and related to the topics discussed during other portions of the workshop. Each session started with a prompt to provide a common structure and to assure that groups were moving at a similar pace in order to maintain relevant and timely educational content each day of the workshop. We reviewed each day what we had done previously. The content from earlier phases were always available and students were encouraged to leverage their work from previous activities in the following ones.

##### Welcome and introductions to innovation processes and STEM (Day 1).

The first day was aimed to welcome participants to the workshop, set ground rules, and explain the concepts we would be focusing on during the design sessions. Students were introduced to concepts such as digital wellbeing, STEM, and STEM identity for girls, as well as the aims of the workshop: innovative ideation and collaborative thinking.

##### Empathize and define a problem (Day 2).

Students were asked to create personas following the prompt: “*Create a fictional character for someone of your age who aspires to a career in STEM*”. Following the first goal of User-Centered Design to empathize with users, we started with an activity on developing user personas. Personas are fictional characters that are developed to set a User-Centered Design team on a common grounding about their users and are typically developed in the early phases of a user-centered design project [40]. Our main priority for the facilitators of this activity was to make this experience a warm-up for getting to know other team members and getting used to collaborating using Google Slides since design activities can provide these opportunities to integrate information and set a positive atmosphere [32].

##### Ideate and sketch (Day 3).

In this session, students were asked to *brainstorm and design a virtual space where teenagers can be their authentic selves*. After building empathy with young social media users, and leveraging our conversations during the workshop about social media usage, we then turned to the next phase of user-centered design: ideation. They were provided with brainstorming rules like building on the ideas of others through a “yes and…”^1^ approach, a rule of improvisation to accept contributions of others, as well as templates to elaborate on their ideas such as a *comicboarding* template [36]. These visual resources included mobile templates, screen layout templates, and interactive components. They were also encouraged to sketch out their ideas on paper. To provide a supportive environment for young girls, we encouraged participants to brainstorm in a private space before sharing their ideas with others in breakout groups; and facilitators assisted groups with technical and social scaffolding.

##### Debrief & present ideas (Day 4).

This session consisted of breakout room preparation and main room presentation. Students were asked to come up with a solution to create a more positive or prosocial online experience for people of their age within their breakout group. To assist them in utilizing their concepts from earlier design phases, they created a collage of their past work *from personas and brainstorming*. Then they were asked to collaborate on designing a new online space as a pitch to an investor. They needed to prepare a presentation that included who they were pitching these ideas to, the size of the online community, who can join, and what technologies exist or must be built to achieve this new online social space. Every student was required to present in the main room final presentation. Google slides were used as an aid to the presentation.

#### 3.3.3 Data collection and analysis.

During the workshop, we collected qualitative and quantitative data about participant’s experiences consisting of: pre-study/post-study questionnaire responses, daily surveys on enjoyment of the activities and comfort level, design artifacts produced during design sessions, and audio/video transcripts obtained from Zoom. We also provided optional online journaling on Seesaw for open-ended spaces to indicate their impressions of activities. To gain an understanding of the diverse range of social media and STEM/computing experiences in our recruited population, we asked about their social media use history, including the platforms they have used and when they began using them as well as their STEM engagement history, e.g., computing camps and clubs.

In this paper, we focus on our qualitative analysis of design sessions occurring in small “breakout” groups. We first conducted inductive analysis of the small group sessions, starting with granular codes and then, through discussion, agreeing on higher-level themes. Two broad areas that emerged were the classification of suggestions and ideas from design partners during the design sessions, and the social collaborative processes that occurred during the sessions. This dichotomy between output ideas and the generative process echoes how other design research projects can be categorized [28]. To classify suggestions and ideas from design partners in our transcripts, we first labeled all contributions to the discussion as “idea suggestion” without evaluating the creativity of the idea. Then, to identify the ideas that contributed to larger insights, we classified original and broader insights as “ah ha moments” which are of a similar scope to the “big ideas” captured in design sessions with children [15, 23]. When describing the artifacts created by the groups in our findings, we describe a thematic categorization of these suggestions and ah ha moments as they emerged in the design sessions. For the collaboration processes, after obtaining our initial codes, we a) reviewed how the intergenerational collaboration framework by Yip et al. [50] for the collaboration strategies and the virtual synchronous model from Lee et al. [34] relate to our codes in the context of relevant frameworks and b) identified gaps specific to our workshop structure population.

#### 3.3.4 Participants.

We had 17 participants from 8 different middle schools across 4 states from the east to west in 3 different time zones (California, Missouri, North Carolina, Massachusetts). They were 2 6^th^ graders, 8 7^th^ graders, 6 8^th^ graders, and 1 9^th^ grader, ranging in age from 11 to 14. Our participants (16 identified as female and 1 identified as non-binary) were also ethnically diverse, 9 identified as Asian, 6 White, 4 Black, and 1 Native American. Participants have been anonymized, and a summary of their demographics is outlined in Table 1. Participants in our workshop had a large range of prior experiences with mobile device ownership and social media usage as reported in Table 1. A sizable number of students had not owned their own smartphone yet. Out of those who were actively using social media, almost all of them signed up at age 11 or younger. YouTube and TikTok were the most frequently reported favorite social media sites. When organizing participants into small groups, we prioritized age and stage in life, that is, we grouped rising 6th graders together, 7th graders together, etc.

## 4 FINDINGS

First, we present the artifacts produced from our design sessions and illustrate our process and activities that led to these creations, and then, we describe the collaborative process that led to the formation of these ideas. These results present our procedures and outputs from a virtual low-tech design workshop with underrepresented early adolescent girls in Tech and ICT careers.

### 4.1 Description of design sessions and resulting artifacts

The design activities were led in small groups, with 3 to 4 students per group and one facilitator. There were five groups total - we refer to these groups according to their color when presenting our results. Groups met during the assigned small group sessions in individual Zoom meetings, as opposed to utilizing the Zoom breakout feature to aid with data collection.

#### 4.1.1 Personas.

**Figure 1:**
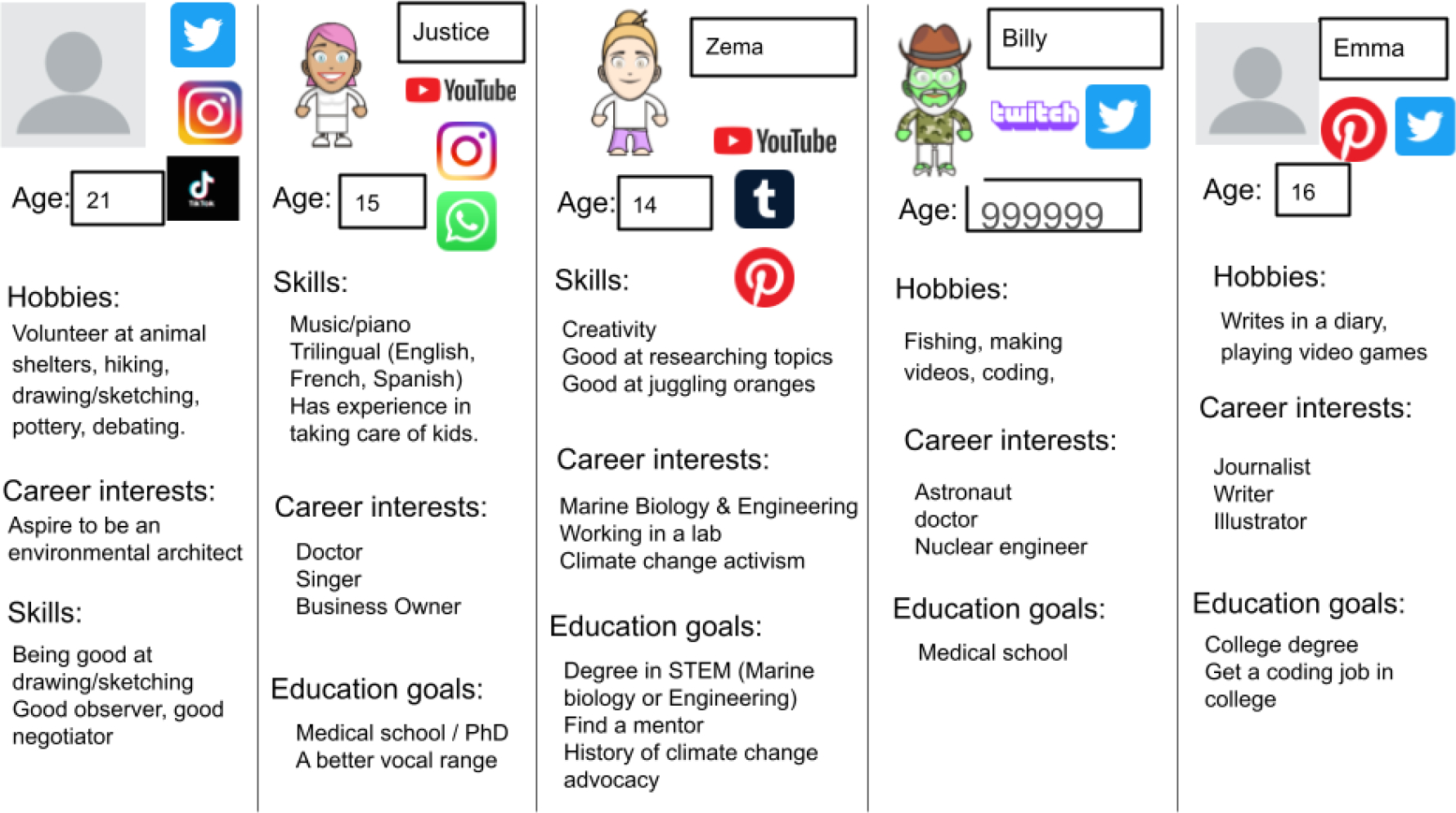
A summary of the personas created by the small-groups during the Personas/Empathy design session.

On the second day of the workshop, students were divided into small groups and were provided with an editable Google Slide document containing the template for their personas. The session started with an explanation of personas as fictional characters representing future users of a digital system [40]. Then we provided a description of the goal which was to “*Create a* ***fictional character*** *for someone of your age who aspires to a career in STEM*.” In small groups, facilitators discussed the guidelines to take a “*yes and*…” stance which is an inclusive approach to building on each other’s ideas, giving a chance for everyone to speak, and an explanation about how to edit the document. The task for this activity was to create a persona using the single-slide template, and in a second slide providing an example of a social media post that this character would post on their favorite platform. We provided resources in appendix slides including examples and graphic icons that could be copied and pasted to the group slide. We also proposed using a website called dpple.me to create the visual characters representing each persona as an outlet for digital manipulation and to support discussion. These sessions lasted 20 minutes and are summarized in Figure 1.

Students created personas covering a wide range of hobbies, skills, career interests, and educational goals. Some characteristics were the subject of extended team-bonding. For example, Zema is good at juggling oranges, which was a spontaneous suggestion from a participant as a skill, and which later resurfaced multiple times through the persona session (such as a useful skill for TikTok) and later as the name of the group’s final project. In all sessions, facilitators took the lead with editing the collaborative Google Slide deck. In some groups, some students also contributed their own edits while others relied on facilitators for edits. Facilitators encouraged participation either by speaking up, and they would type their contributions, or by encouraging the usage of analog design tools (pen and paper) and then sharing those sketches. Starting with this activity, and prioritizing the comfort of participants, team building, and technical warm-up, allowed us to then scaffold the activities using Google Slides. At the end of the session, one student commented that: “*I really like activities like the persona one and I think more of those would be cool*” (P17, blue group, 13 years; day 2 survey).

##### Building block suggestions.

Many suggestions proposed during the personas sessions were requests for specific color choices or naming alternatives for personas. These suggestions served as “social glue” or conversational prompts to encourage student participation and create a welcoming environment for idea generation. We called these building block suggestions because they were encouraged and valued as part of opening the door to participant’s creative thinking but did not necessarily lead to insightful designs about novel social systems.

#### 4.1.2 Brainstorming.

**Figure 2:**
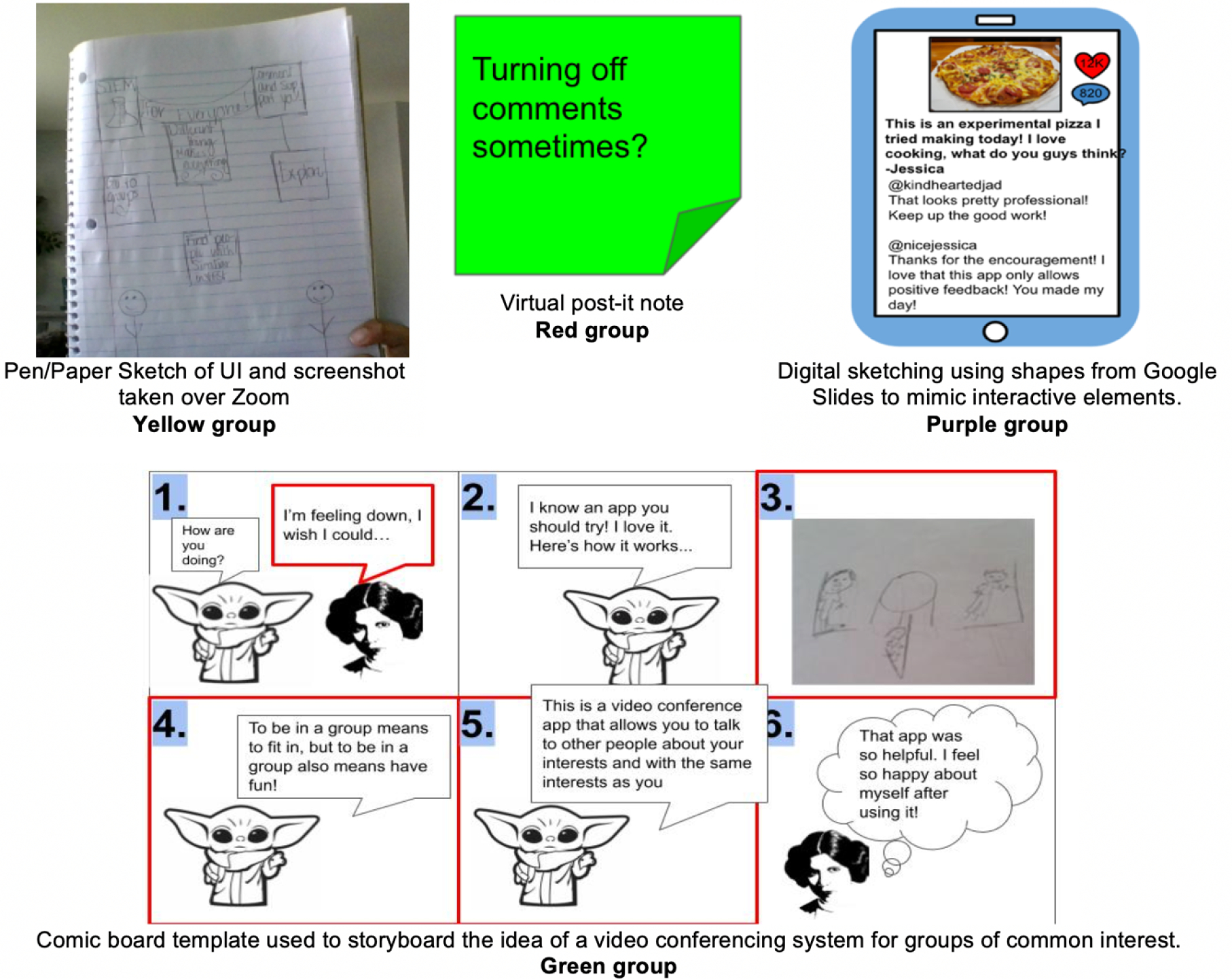
Example brainstorming artifacts created by different groups during the Brainstorming session.

On day 3 of the workshop, we led brainstorming design sessions with the same small groups as the personas to keep the rapport between the students and the facilitators. The brainstorming session lasted 35 minutes and was divided into two parts: divergent thinking in which students were encouraged to think of “wild” and “outside the box” ideas; and then a convergent thinking exercise to design a sketch UI for a cohesive idea. In the brainstorming session, students were provided 3 template slides to encourage ideation: virtual post-it notes, blank pages to upload sketches, and a comic board template. Figure 2 illustrates outputs from this session.

##### Inspiration from offline experiences.

Participants described offline experiences as analogies for online social spaces or as ideas specifically answering the brainstorming prompt: “*how might we help teenagers explore their interests and be their authentic selves online?*” We observed that participants focused more on answering the aspect about encouraging teenagers to be their authentic selves than a design idea for the online environment. These offline experiences were focused on connecting with interest-based groups: “*Talking to people with the same interests as themselves*” (P1, 12 years; Seesaw) and provided ideas about different types of face-to-face experiences catered for self-exploration for teenagers “*Like go to certain summer programs that will further explore your interest*” (P10, 13 years; Purple group, Brainstorming).

##### Encouraging positive end-user behaviors.

In the Seesaw activity and some brainstorming sessions, we observed many ideas pertaining to suggestions for modifying or encouraging end-user behaviors. For example: *“creating smaller communities inside of social media apps to connect with even more people that you have a lot in common with”* (P17, 13 years; Seesaw), *“don’t post negative things because you don’t want people coming at you”* (P6, 11 years; Red group, Brainstorming).

#### 4.1.3 Final project ideas and pitches.

**Figure 3:**
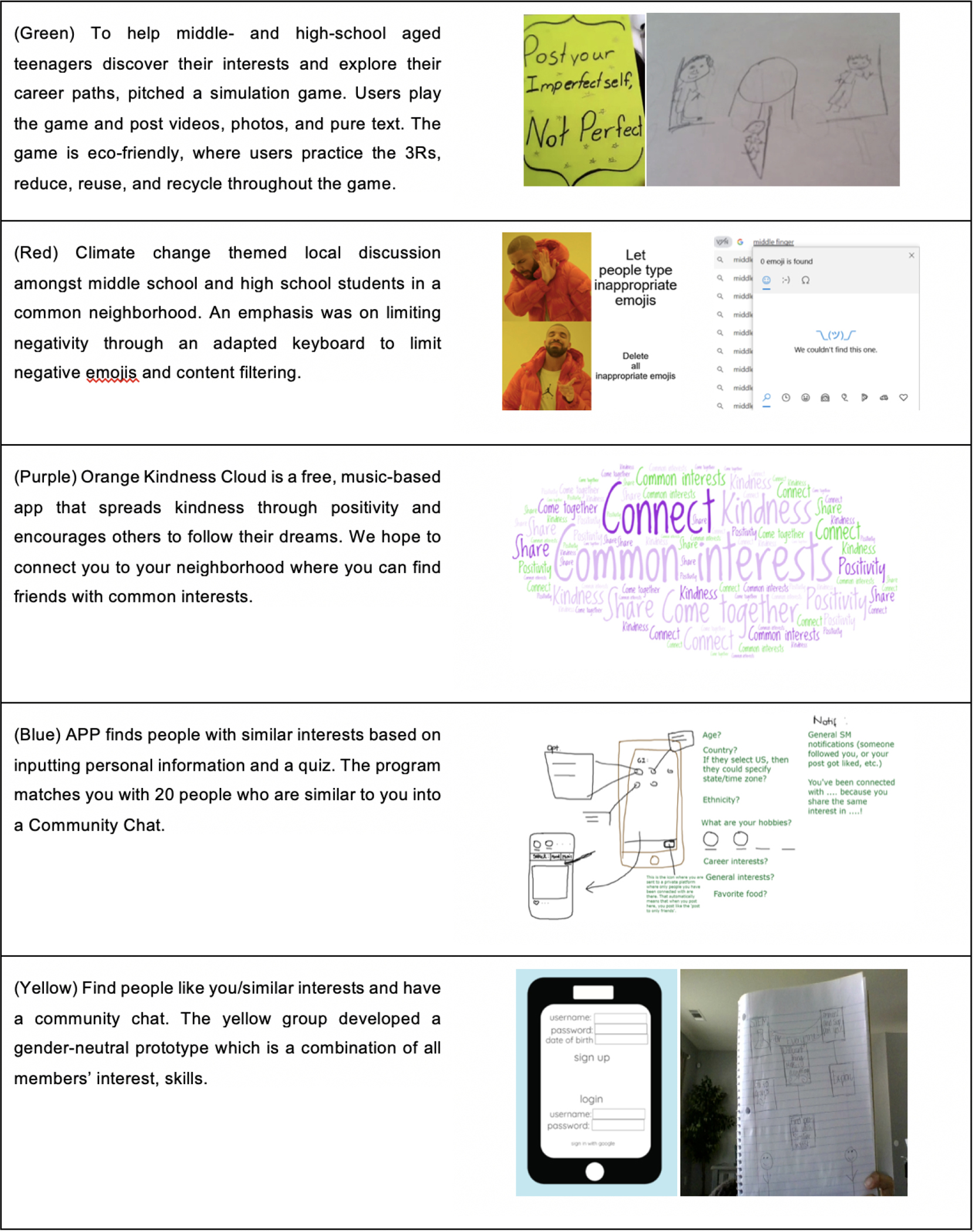
Summary of the final project pitches proposed by each group.

We present the projects that culminated from this workshop as a capstone showcase for the collaborative design process we fostered throughout the week. These ideas were developed on Day 4 (the final day) of the workshop after discussions about social media usage, digital wellbeing, design process and innovation; see Figure 3. Students were encouraged to bring ideas and creations from previous days into their final projects, and were encouraged to discuss with each other on idea convergence to present one cohesive idea. These project ideas do not represent a comprehensive design space of all possibilities for this prompt, but rather illustrate themes that were meaningful for this population and demonstrate the feasibility of the scope for projects conducted in a synchronous online environment about designing novel ICT experiences. Our design prompt for this session was “*Creating an online community or app that enhances positive or prosocial interactions within teens*.”

The “big ideas” [23] lead to insightful creative moments and more in-depth exploration of features and mechanisms for communication technologies that occurred during the final project session. Amongst the ideas proposed during the brainstorming session and final projects, we noted four themes emerging from the ideas of participants that were specifically about novel online social interactions and social spaces.

Across these final projects, most groups prioritized having positive interactions in their novel social spaces. They proposed ideas pertaining to *community guidelines and moderation* of social applications. The purple group created a music-based mobile application whose motto was to “spread kindness through positivity and encourage others to follow their dreams.” To maintain harmony within the space, students suggested an algorithmic design that monitors negative comments and removes them from the feed. Similarly, in the red group’s climate-change themed platform, negative comments or emojis were not allowed.

Multiple projects were targeted to *local communities*. For example, the purple group’s music app was targeted for a local neighborhood and participants envisioned that a part of the user recruitment plan would include reaching out locally through “Spreading the word (door-to-door), newspaper, advertisements in stores.” In addition to ideas centered on interest-based groups, this focus on local communities indicates untapped areas in existing ICTs for hyperlocal connections that may be particularly appealing to young users.

Two groups, green and red, devised online social spaces to focus on *social issues*, such as environmentalism and climate change. The red group proposed a platform where teens could come together to discuss climate change while only sharing positive interactions. Similarly, the green group suggested a game-based social platform where kids and teens could learn eco-friendly habits through the game while sharing pictures, videos and thoughts on the social aspect of the application.

*Lastly, finding like-minded individuals and exploring novel mechanisms to connect with others* was a central theme for the blue and yellow groups final projects. The blue group designed an application that used a quiz to match people with similar interests. Students in the blue group offered a broad range of options to connect with peers such as private chats, a general feed and community groups. The yellow group designed a community chat for peers with similar interests through a gender-neutral prototype that combined all members’ skills and interests. All these groups highlighted different ways to build a community as well as ways to have more positive and prosocial interactions online.

### 4.2 Collaborative processes

We analyzed the collaborative practices occurring between facilitators and participants, and amongst participants, during the three design sessions of personas, brainstorming, and the final pitch project. High-level themes emerged including strategies employed by facilitators for leading the design process, strategies for encouraging idea elaboration, and student-led ideation. We present our analysis of collaboration strategies according to the Yip et al. framework [50] and specifically describe the unique strategies from our workshop related to the virtual context with middle school girls in building rapport. We also contextualize the types of online interactions we observed following the Lee et al. categorization of virtual synchronous interactions with children: Meeting, Sharing, Collaboration, and Design [34], and describe the digital ecosystem of tools used during our workshop for interactions with early adolescents. We noticed different strategies emerge between contexts (e.g., instances, blocks of time) in which there was more active participation from participants as compared to those with more passive participation. We summarize our findings in Table 2

#### 4.2.1 Facilitation for guiding the participatory design process.

In Yip et al., facilitation is defined as “how much support and mediation takes place between the adults and children” which might include “organizing and managing the flow of the co-design session, leading the group discussions, and summarizing the group’s ideas” [50]. In our design workshop, we noticed many instances of facilitation which provided the structure for the activities undertaken. Facilitators were generally responsible for leading these sessions which included managing the structure of the activity, time management, technical support, and managing turn-taking between participants.

#### 4.2.2 Using different digital channels than verbal communication to encourage participation.

In more passive engagement contexts, such as the first session in which design partners were getting to know each other, facilitators employed many strategies to encourage participation in ways that felt most comfortable to participants. When participants were not eager to participate verbally, facilitators leveraged ways for contributions to occur through the different online channels of communication and collaboration tools. For example, one facilitator encouraged participants to share their ideas with them through any means of preference including Zoom chat or their personal email: *“Now’s your time to shine. You can draw something or just shout out ideas you have of how this person may look like. You can honestly just grab a piece of paper and pencil and then maybe, here, I could drop of in the chat my email, so that you could send any ideas, you have to it”* (Blue group, Personas). Collaborative tools provided an opportunity for synchronous participation, such as Google Slides with its affordance of presence indicators to note where other design partners were modifying a slide. As a facilitator noted out-loud: *“it looks like maybe P7 is working on slide three on the emojis”* (Red group, brainstorming).

#### 4.2.3 Using digital tools to facilitate passive endorsements.

To adapt to a situation of passive engagement during a brainstorming session, when a facilitator wanted to encourage idea convergence through group consensus, the facilitator adapted to any lack of verbal participation by proposing to use Google Slides to endorse the ideas that participants wanted to move forward with: *“What do you think would be an interesting direction to go in? How about we do some votes on these. Could you make a little box with your name in it so like, for example, this would be me. And then you just put your name on whichever […] ones you’re the most interested in. […] Just to give you an example of how to do that. And if that works for you P6 and P5 I’d love to also see what you’re thinking, and thanks for putting those votes in P7”* (Red group, Brainstorming).

#### 4.2.4 Facilitating elaboration for encouraging idea generation.

During Yip et al.’s *elaboration phase*, facilitators and participants engage in “generating and mixing ideas together” [50]. During instances of *elaboration*, facilitators were often prompting participants to think about their ideas and develop them. They also provided additional support for thinking outside of the current context of design, and provided encouragement for divergent thinking such as encouraging “wild ideas”. Younger students had more challenges generating wild ideas, facilitators we more actively involved groups with younger participants. When students were engaged in elaboration, they discussed their ideas with their group. We observed instances of balanced engagement between students and facilitators, often occurring through a facilitator prompt and students answering.

#### 4.2.5 Placing emphasis on hearing everyone’s voice.

In one instance, where a facilitator was trying to encourage idea generation during brainstorming but participants were not engaging, they changed the direction of their prompting and asked students about their own experiences on social media. When asked more about their favorite platforms, one student answered “TikTok, Instagram and Snapchat”, which then led the facilitator to ask what they like about them, and the student answered: “they’re kind of relatable. You can connect thoughts with people, you don’t have to know them but you’re feeling the same way” (Blue group, Brainstorming). These types of prompts were attempts to appeal to participants’ own experiences to make a meaningful bridge to the task at hand. Through this exchange, the student proposed the insight that social media platforms allow them to connect with others they don’t know based on common interests. This exchange ultimately paved the way for the final project on the idea of finding similar others through filling out an app-based quiz and being matched with others in a community chat.

#### 4.2.6 Providing scaffolding towards developing solution ideas.

In contrast to contexts with passive participation in which facilitators employed a variety of multi-channel strategies and prompting techniques, facilitators also played a role in supporting elaboration in active participation contexts. As we described previously, participants envisioned many different types of ideas ranging from basic, “building block” ideas to more insightful ones. In order to achieve these in-depth insights, facilitators provided support to think further about their ideas. In this exchange, a facilitator prompts a participant to relate an offline experience to an online one:

Facilitator: Can you explain it a little bit of what you’re drawing here?

P1: So the people that are kind of like in a group together or a club, you know they talk about their interest in. Guess you can say science, or just STEM.

Facilitator: Is this an online platform?

P1: No.

Facilitator: If this were an online platform, do you have any ideas how this would look like?

P1: A bunch of video cameras. Video chatting. Kinda like, you know Zoom or Google Meet.

(Green group, Brainstorming)

In this example, the participant had created a sketch using pen and paper, and shared it with the facilitator over the camera on Zoom. The sketch illustrated a group of people sitting around a table. Through the guidance of the facilitator, the student is led to an insight about the technical implications for creating an online experience that could support the socialization they were envisioning for a positive offline experience.

#### 4.2.7 Idea merging using private and public/shared spaces.

In the purple group, students shared ideas that they individually had developed before the session on Seesaw, or their own sketching tool, to then bring them to the group. They each shared their ideas which represented vastly different concepts: *“On my idea it’s like kind of crazy, basically they ask what you’re interested in and they automatically generate a song for you”* (P9); *“you talk to experts, where you could be like an expert per se”* (P8); and *“You put anything like a picture of what you did that day or like writing something that you’re very passionate about. People have to leave positive comments. Whereas if you don’t make a positive comment, they will kick you out of the app or something”* (P10).

Following the sharing of their ideas, the facilitator then led the group to think about how these ideas could be merged to create a socio-technical solution for positive online social spaces. An excerpt of this merging process went as follows:

Facilitator: What do you think is a mutual point for all three of you?

P10: I think we all had something that involves posting something and then, like others, will give you a response. Like for songs, you post something and others respond like.

P9: You can basically combine [that with the two other ideas]. So basically, if you generate your song, you can post it on to the app and then they can comment positive things about it.

(Purple group, Brainstorming)

In this interaction, we observe that the private refection to develop their individual ideas, (e.g„ using Seesaw) was central to enabling this idea generation discussion. Even though a primary focus of the digital ecosystem that supports collaboration in virtual contexts are tools that provide shared spaces of visibility and presence, we found the support of these private refection spaces essential in the design process as well.

The different strategies of elaboration we observed that occurred in this synchronous virtual context relied on the direct guidance of the facilitator, regardless of whether participants were more or less active participants. In contrast to instances observed in in-person settings where children may be ready to engage with each other [50], we found that in our virtual settings facilitators were initiators of elaboration discussions.

#### 4.2.8 Design-by-doing.

Design-by-doing refers to instances when “design activities take place” [50]. Yip et al. notes that there is not always a clear separation between moments of design-by-doing and those of elaboration, both part of ideation. Elaboration pertains more to the active discussion of ideas while design-by doing refers to the manipulation of design tools. In a synchronous virtual design session, we found the separation to be even more blurry since the elaboration discussion is supported by a virtual tool. Thus, we note instances where design tools, those going beyond the sharing and collaboration tools used during elaboration, as design-by-doing.

In some groups, students led design conversations and took control of digital tools to express their ideas and discuss them amongst each other. In the purple group, students used different external tools based on ones we presented during the workshop or ones they already knew about to express their ideas and contributed to our shared Google Slides: *“Can I share my screen, because I digitally did it on Procreate?”* (P8, 14 years; Purple group, Brainstorming). This participant then describes her idea: “*So it’s just a rough sketch because I was doing it kind of fast. But, so I gave the option that to answer a question or to add and so it’s like the daily question on the top and then any user can post a question. And then I just did a sample question like what have you been doing this past month, and then you can reply anonymously, or like the post. So taking a drawing or just a bunch of anonymous replies, so you don’t need to worry about anyone else, like seeing what you don’t want to see.*”

In groups in which students engaged more passively, facilitators prompted idea elaboration through using the visual support of a design tool, such as asking about simple styling choices for building the persona character during that initial activity.

#### 4.2.9 Setting a positive atmosphere.

Building a close relationship during PD is also an important aspect of intergenerational co-design [50]. Through many of the strategies described above, facilitators built rapport in the virtual context and fostered a positive environment. We noted one specific strategy employed by a facilitator in an active group. In this group, students contributed through verbal exchanges and had many ideas. The facilitator suggested that they each generate their own ideas, in a private space, and conduct work in parallel for a few minutes. The facilitator then provided support and set a warm environment for students to do their work by playing music, and asking for input from the group: “*While you’re sketching let’s listen to some music. Any song requests? Any favorite songs? Anything else I’m gonna play some very old song. Let’s see. [plays music while they work]*” (Green group; Brainstorming).

#### 4.2.10 Individual trajectories of growth.

We present three vignettes of participants to demonstrate their trajectory of growth. Each vignette presents girls in different grade levels. While they are not representative of all girls in their grade, they illustrate some particular differences in comfort levels and how facilitation strategies contributed to their experiences.

P7 was one of the youngest participants - a Chinese-American student (aged 11) entering 6th grade. She participated in the red group. Her favorite part about the workshop was the “Breakout Rooms”. She contributed the most in the first breakout session, by answering prompts from the facilitator and adding in-depth insights. In the second session, P7 was more passive and contributed primarily through Google Slide. In the final session, she contributed more frequently such as assisting the facilitator by linking external online resources, explaining how she used a digital tool, developing on ideas through prompts from the facilitator and volunteering her own. In the post-study survey, she described the workshop as “*Fun and Easy, simple and amazing.”*

P15 was a quiet Chinese-American student (aged 12) entering 7th grade. She participated in the blue group. Her contributions progressed with each design session. She contributed many in-depth ideas to the final project such as the idea to fill out a survey to see what a user is interested in; sort users into like-minded communities; automatically generating groups; setting limits on how many people one can follow; and adding a hyperlocal component so that users can connect with others close by. She felt positive about the experience when asked whether it was a safe environment: “*Yes, I felt like it was a safe place because everyone was very nice and supportive to each other, and there were no mean or negative comments and remarks*.” Her favorite part about the workshop was “*doing the last project, which was designing our app that helps people find their interests*.”

P12 was a vocal African-American student (aged 13) entering 8th grade. She participated in the yellow group. She was an active social media user, owning a smartphone and using social media since she was 9 or younger. She was an active participant throughout the workshop, contributing to large-group discussions. In her small group, she increasingly participated in each session and felt positive about the experiences: *“I think the breakout sessions were great - it was a way for me to be able to get to know new people as well as be able to interact with people without being nervous.”* In the second session, she took initiative for directly modifying a Google Slide to sketch her idea and then asked the facilitator to show her design to the group for feedback. She was invested in her initial brainstorming idea, and expressed her interest in continuing this idea as the final project. She also had the confidence to take the lead in presenting the first slide during the final group presentation.

#### 4.2.11 Summary of Collaborative Processes.

Collaboration strategies varied by context, such as design phase and participant engagement. Facilitators managed the structure and dynamics of sessions. In groups with minimal discussions, facilitators encouraged participants to use different digital platforms to increase contribution, and used digital tools for endorsing ideas. Facilitators initiated elaboration processes and placed an emphasis on hearing everyone’s voice, scaffolding collaboration, and idea merging. There was a balanced contribution during Design-by-doing, for example students in active groups used screen sharing to discuss design ideas. Relationships between facilitators and participants were managed by facilitators who set the atmosphere. Individual trajectories of growth indicate how these strategies resulted in engagement and personal growth across participants from different racial/ethnic backgrounds, ages, comfort levels, and prior digital experiences participating in these collaborative processes.

## 5 DISCUSSION

### 5.1 Idea development from building block suggestions to “big ideas” through collaboration (RQ1)

#### Big ideas:

We noted four themes emerging from the final projects for positive online social interactions and social spaces: *community guidelines and moderation* of social applications; multiple projects targeted to *local communities* such as local neighborhood; ideas centered on interest-based groups through *finding like-minded individuals and exploring novel mechanisms to connect with others*; and a focus on addressing *social issues*, such as environmentalism and climate change, through online social applications. Even though photo-based applications are topics of particular interest for middle school girls [14], none of the projects relied primarily on photo-based or photo-sharing mechanisms. Similarly, while non-White students were significantly more interested in posting something that would get a lot of views [14], participants in our workshop did not focus on reactions mechanisms. Instead, ideas demarked themselves from common social media paradigms such as a music-based mobile application that would create a song based on input from the user; and fostering more intimate connections rather than large-scale broadcast by facilitating hyperlocal connections. Through our workshop structure, including the topics we discussed around digital wellbeing, our guest speakers motivating young girls in tech, and our design pitches around *being their authentic selves* and *positivity* online, we found that our population of diverse middle school girls expanded beyond traditional models of online social interactions to propose ideas that could foster more supportive, safe, and engaging spaces for them.

#### Building block ideas:

Not all ideas produced by participants pertained directly to features or mechanisms of social systems. This matches prior work in design sessions comparing outputs between younger children and adolescents where younger children might propose more aesthetic types of ideas [15]. When designing with middle school students, it might be more common to observe these types of building block ideas with younger students, such as 11 or 12 year olds in 6^th^ grade, than older students. To be inclusive of age differences, these types of answers should be encouraged as part of students’ contributions. In our remote context, we observed that many of these initial ideas, such as *encouraging positive end-user behaviors* and *inspiration from offline experiences*, were written in individual Seesaw activities that happened before the brainstorming session.

#### Social media to explore computation.

This topic was also fruitful as the basis for computational exploration and creativity with this population as it exposed participants to many computational concepts. They thought about and heard others’ ideas on a broad range of technical topics including robots, algorithms, or hardware devices. For example, through discussions of digital wellbeing, positivity, and empathy, participants focused their solutions on encouraging more positive interactions, such as removing negative content algorithmically or preventing posters from adding negative content through modified keyboards. Their design ideas were of particular salience to their age demographic such as intersecting social interactions around positivity in the context of discussing environmentalism.

To answer RQ1, early adolescent girls produced design ideas focused on community-oriented and societal issues rather than media-centric applications such as photo-sharing mechanisms. We also found that many ideas suggested were *building block* contributions: they did not pertain directly to innovative ideas but were contributions to the tasks of the group. By creating a supportive environment for building block ideas to emerge, participants were able to have their voices heard in the design process. Ultimately, the goal of the workshop was not a final working social prototype, but rather fostering collaboration and meaning-making and through the design activities. Through engaging with middle school girls as experts in their needs around social media, we surfaced topics of concern and conceptualizations for more positive spaces for them.

### 5.2 Digital tools and strategies for synchronous virtual design with middle school girls (RQ2)

*A digital ecosystem of multi-modal forms of communication and self-expression* were effective during our workshop. Using Zoom as our video-conferencing platform provided constant communication through audio and video feedback, while allowing students to maintain control over when to show their videos or not. We found that most of the participation from students occurred through verbal communication on Zoom. Some were also highly active in directly editing the Google Slides but most relied on the facilitator to make those edits. We noticed less engagement in the small group Zoom chat function than we did in the large-groups, which may have reflected less comfort in participating verbally in the whole group sessions. We also observed instances of participants utilizing external tools and presenting them through screen sharing, such as the drawing tool Procreate or a web application for creating a word cloud infographic. Encouraging the usage and exploration of these external tools can be a way to encourage peer-instruction, collaboration, and personal agency in the design process. These tools introduced new challenges that are less salient for in-person design workshops. Facilitators noted instances when they weren’t aware of what participants were doing with lack of feedback (e.g., cameras of). Facilitators also faced some challenges in supporting students’ technical issues since they could not directly view participants’ screens, or spotty wifi limiting the ability to hear each other properly.

#### Facilitators were essential in coordinating online activities.

The facilitators navigated similar practices to those observed during in-person sessions such as assisting participants in expressing their views, promoting reflection and problem solving, and resolving consensus and disagreements [18]. In our distributed synchronous context, we also observed ways in which the mediation of technologies utilized for meeting, sharing, collaboration, and design [34] created new opportunities for facilitators to assist their team through resolving technical issues or using passive endorsements, such as name labels for voting, to encourage participation. We found that our facilitation methods that were conducive to early adolescents aged 11–14 contained aspects of both Yip et al. [50] and Lee et al. [34] frameworks, resulting in identifying intersections between both approaches. For example, in situations with low engagement, *facilitation strategies* leveraged *collaboration tools* such as encouraging the use of different digital platforms to encourage contributions, or for idea endorsements. In *elaboration* processes *meeting tools* allowed facilitators to place emphasis on hearing everyone’s voice, and suggesting idea merging.

#### Size & composition of breakout groups.

We found many positive comments about the small group sessions in our daily questionnaires which resulted in experiences of mutual learning. Despite different levels of engagement in the small groups, these findings were consistent throughout suggesting that facilitators were able to adapt their team building and rapport building strategies depending on the needs of each group. We noticed less participation in groups composed of younger participants (e.g., tweens) than those that were older (e.g., early teens), and more cases of younger students refusing to show their cameras, participate verbally, or deflecting questions. However, we did not notice more conflicts or disagreements in these groups as was noted in [15]. We observed facilitators propose a range of techniques to encourage participation, and given the positive feedback from participants about their experience in small groups, we suggest these techniques as effective for small group discussions with this age group. Future work could elaborate on participation and confidence-building strategies and scaffolding for the youngest of our groups (11 and 12 year olds who are newly entering middle school or who did not yet have a smartphone).

To answer RQ2, facilitators were essential for the design activities and they oversaw the coordination of the digital tools we used for meeting, collaborating, designing, and sharing contributed. In a remote design workshop with middle school girls, particularly when the goal of this setting is to enable participation and create a supportive environment, we found that small breakout groups led by facilitators were appreciated and resulted in many instances of mutual learning, collaboration, and encouragement.

Developing novel ICT spaces was engaging to our participants - underrepresented middle school girls - in a safe virtual space where their gender and race were the majority rather than the minority. We found that this topic was particularly amenable to the virtual design context via: a) positive, comfortable, and interactive online space with other people like them to see what is possible in virtual contexts, b) an opportunity to discuss digital wellbeing and their initiative in using social tools, and c) a context that provided them control and agency to manipulate their own presence and participation in the workshop.

## 6 LIMITATIONS

The results from this study should be contextualized by the population of our workshop participants and our recruitment strategies, which are standard for this type of research. Our purposive sampling strategy was first advertised to a diverse school community where we had an existing partnership and, secondly, we used word-of-mouth to reach hard-to-find youth. Our participant selection criteria was based on prioritizing heterogeneity [39] to include those underrepresented in the current design of technology (e.g., race, socioeconomic status, gender). This recruitment process resulted in a geographically-dispersed population. We chose to conduct the workshop during a mid-day timeframe that would be equally suited for East Coast to West Coast participants.

As these findings are based on 17 participants, the findings need to be replicated in future work with more participants of a similar age demographic. Our findings about resulting artifacts, collaborative technologies, and our digital ecosystem may not be exhaustive of all possibilities observed in design sessions with middle school participants. However, the learnings we obtained about collaboration during the workshop could be applied with other middle school populations we specifically targeted, whose voices are underrepresented in the current design of technology. Our paper describes in detail our workshop design process so that future researchers can employ our activities and analysis methods which may be applicable to early social media users.

## 7 CONCLUSION

This paper presents our results and insights from a synchronous virtual design workshop with 17 ethnically diverse, and geographically-dispersed middle school girls (aged 11–14) to co-create novel ICT experiences. We leveraged social media as a domain of high relevance in the lives of most young adolescents to encourage collaboration and self-confidence as creators of digital technologies. Our participatory workshop centered on social media innovation, collaboration, and computational design. The culminating design ideas of novel online social spaces focusing on positive experiences for adolescent girls included different types of ideas from building blocks, to offline experiences, to solutions for online prosocial spaces such as algorithmically removing negative comments. We observed facilitators used a variety of strategies to guide participants through the process and ideation of these ideas, employing different strategies based on the engagement levels within their small groups. The outcomes of the workshop were overall positive, participants particularly enjoyed the small breakout groups and final projects. We found that utilizing social media as a domain for computing exploration with diverse adolescent girls was effective and motivating, and that our virtual synchronous workshop was a comfortable environment for this self-exploration. Our findings demonstrate the feasibility, utility, and promise of incorporating participatory design principles for activities embedded within informal virtual workshops related to STEM or ICT with students of this age and background.

## Figures and Tables

**Table 1:** Demographics, smartphone usage, and social media usage of workshop participants

Design group	ID	Age	Race	State	Age of first smartphone	Age first joined SM	Favorite social media (SM) sites
Green	P1	12	Native American (e.g., Cherokee, Apache)	NC	9 or younger	9 or younger	YouTube
	P2	12	Asian/Pacific Islander	MA	Not yet	–	YouTube
	P3	12	Black/African American/Haitian/West Indies	MI	9 or younger	11	YouTube
Red	P4	11	Black/African American/Haitian/West Indies	NC	9 or younger	9 or younger	Snapchat, TikTok, YouTube, House Party
	P5	12	Asian/Pacific Islander, White/Caucasian	CA	Not yet	11	TikTok, Discord
	P6	11	White/Caucasian	MA	10	9 or younger	Snapchat, TikTok
	P7	11	Asian/Pacific Islander, White/Caucasian	CA	Not yet	9 or younger	TikTok, YouTube
Purple	P8	14	White/Caucasian	MA	14+	13	TikTok, YouTube
	P9	13	Asian/Pacific Islander	CA	Not yet	11	YouTube, Discord
	P10	13	Asian/Pacific Islander	MA	Not yet	N/A	None
Yellow	P11	12	Asian/Pacific Islander	CA	12	9 or younger	YouTube, Discord
	P12	13	Black/African American/Haitian/West Indies	NC	9 or younger	9 or younger	TikTok, YouTube
	P13	13	Asian/Pacific Islander, White/Caucasian	MA	11	11	Snapchat, TikTok
Blue	P14	12	Black/African American/Haitian/West Indies	NC	9 or younger	11	TikTok, YouTube
	P15	12	Asian/Pacific Islander	CA	Not yet	9 or younger	YouTube, Discord
	P16	12	Asian/Pacific Islander	CA	10	N/A	YouTube, Other
	P17	13	Black/African American/Haitian/West Indies	MA	–	–	–

**Table 2:** Model of collaboration practices observed in a distributed participatory design workshop intersecting the PD intergenerational practices highlighted in [50] and the online interactions mediated by digital tools from [34]

Collaboration dimensions (from [50])	In-person emphasis (from [50])	Virtual emphasis	Instances of virtual collaborative interactions	Online interactions mediated by digital tools (from [34])	Digital tools used in our workshop to support online interactions
**Facilitation**	Facilitator-led	Facilitator-led	Different channels	Sharing, Collaboration	Zoom chat, email, Google Slides
			Passive endorsements	Collaboration	Google Slides
**Elaboration**	Balanced	Facilitator-led	Placing emphasis on hearing everyone’s voice	Meeting	Zoom audio
			Scaffolding to solution ideas	Meeting	Zoom video, audio
			Idea merging with	Meeting,	Seesaw, Google
			private/shared spaces	Sharing + Private	Slides, Zoom audio
**Design-by-doing**	Balanced	Balanced	Screen sharing for discussions about design ideas	Meeting, Sharing	Zoom screen sharing, audio
			Building block ideas with simple tools	Meeting, Design, Sharing	Zoom screen sharing, audio, creativity tool
**Relationship**	Distance between design partners	Comfort managed by facilitator	Team building (music)	Meeting	Zoom audio

## CCS CONCEPTS

• Human-centered computing;; • Human computer interaction (HCI);; • HCI design and evaluation methods;

## Footnotes

1
https://www.secondcity.com/how-to-say-yes-and/

## A APPENDICES

**Table:** A WORKSHOP AGENDA

**Day 1**	1:00 PM **Welcome**	Housekeeping, guidelines, and ice breaker
	1:15 PM **Introduction**	Introduction to the purpose of the workshop and concepts of *Digital Wellbeing* and *STEM Identity*
	1:30 PM **Guest Speaker**	Gitanjali Rao - *Time* Magazine’s Kid of the Year
	2:00 PM **Seesaw Activity**	STEM Identity Charts
	2:20 PM **Breakout Group Activity**	Promotion: Design a social media campaign for STEM identity
	3:00 PM **Innovation in Tech**	Talk about innovation, building apps, and the engineering process. Activity: Think about problems that can be solved by building apps.
	3:25 PM **Closing Survey**	
**Day 2**	1:00 PM **Icebreaker**	Complete career survey on Roadtrip Nation and discuss the results
	1:15 PM **Protection and Self-regulation**	Early experiences with social media: How did you learn about it?
		The balance between self-regulation and creating meaningful experiences
	1:30 PM **Design Activity**	Empathy with users: create fictional characters about teenagers with STEM interests and use social media for STEM aspirations
	2:00 PM **Popularity and Validation**	Discuss awareness of how others affect us online; Social validation, fear of missing out; Mindfulness practices to reduce anxiety
	2:25 PM **STEM Identity Intern panel**	4 interns reflect on their STEM identity, followed by Q&A
	2:20 PM **Seesaw Activity**	Reflections of future self
	3:25 PM **Closing Survey**	
**Day 3**	1:00 PM **Icebreaker**	Kahoot Activity
	1:15 PM **Positivity and Authenticity**	Discuss Realistic vs. ideal selves; Crucial Cs -- Connect, Capable, Count, Courage; Power of images and representation; Raising social awareness
	1:40 PM **Seesaw Activity**	Post “makeovers”: re-designing social media posts to promote positivity
	2:00 PM **Design Activity**	Brainstorming and design activity: How might we help teenagers explore their interests and be their authentic selves online?
	3:00 PM **Design session debrief**	
	3:25 PM **Closing Survey**	
**Day 4**	1:00 PM **Workshop Discussion**	STEM Identity social media campaign result revision
		Feedback for the workshop.
	1:30 PM **Demos**	Prototyping tool demonstrating ideas from Day 3 and Scratch demo
	2:00 PM **Design Activity**	Pitch a more positive or prosocial online experience for youth
	2:40 PM **Pitch Presentations**	Presentations of digital storytelling pitch projects.
	3:25 PM **Closing Survey**	
